# Colorectal cancer in hyperplastic polyposis syndrome: In search of the polyp of origin

**DOI:** 10.1186/1897-4287-9-S1-O7

**Published:** 2011-03-10

**Authors:** Christophe Rosty, Michael D Walsh, Neal I Walker, Mark A Jenkins, John L Hopper, Kevin Sweet, Susan Parry, Daniel D Buchanan, Joanne P Young

**Affiliations:** 1 Department of Molecular and Cellular Pathology, University of Queensland Centre for Clinical Research, Herston, QLD 4006, Australia; 2 Familial Cancer Laboratory, Queensland Institute for Medical Research, Herston, QLD 4006, Australia; 3 Envoi Pathology, Herston, QLD 4006, Australia; 4 Centre for MEGA Epidemiology, School of Population Health, University of Melbourne, Carlton, VIC 3053, Australia; 5 Division of Human Genetics, Ohio State University, Columbus, OH 43221, USA; 6 Department of Gastroenterology, Middlemore Hospital, Auckland 1640, New Zealand

## Background

Hyperplastic polyposis syndrome (HPS) is a colorectal cancer (CRC) predisposition of unknown genetic aetiology that is characterised by the presence of multiple serrated polyps throughout the colon, and an increased risk of having a first-degree relative with CRC [1-3]. Though there is a trend for association between CRC and a higher number of polyps, patients with at least one colonic conventional adenoma have an increased risk of CRC compared to patients without conventional adenoma (odds ratio: 3.6) [2]. HPS was first thought to represent the familial model for the serrated neoplasia pathway, as an analogy to the familial adenomatous polyposis syndrome for the adenoma-carcinoma pathway. However, CRC in HPS patients appears to arise from both conventional adenomas and serrated polyp subtypes. To further define the carcinogenesis of HPS related colorectal neoplasia, we sought to characterise the histological features and the molecular alterations of the different types of benign polyps arising in HPS patients both in contiguity with and remote from CRC.

## Methods

A total of 151 patients diagnosed with at least 5 serrated polyps outside the rectum were recruited from high-risk genetics clinics. Polyp counts were extracted from colonoscopy reports. Polyps, including contiguous polyps, and CRCs underwent pathology review and testing for *KRAS* codon 12 and 13 and *BRAF* V600E somatic mutations.

## Results

CRC was identified in 56 patients (37%) with 31/56 (55%) being females. The mean polyp count in patients with CRC was 58, and their mean age was 52 years. A total of 65 CRCs were available for analysis. Where site was known, most CRCs 43/61 (71%) arose in the proximal colon; however, only 19/58 (33%) of CRCs demonstrated a *BRAF* V600E mutation. Somatic *KRAS* mutations were less frequent at 9/48 (19%). Contiguous polyp was seen in 16/53 (30%) evaluable CRCs, and of these 4 (25%) were tubular adenomas, 5 (31%) were tubulo-villous adenomas and 1 (6%) was a villous adenoma. Overall 10/16 (63%) showed conventional adenomatous morphology whereas 6/16 (37%) had serrated morphology. *BRAF* mutation was present in 5/16 (31%). CRC was present in more than one-third of clinic-based individuals who presented with multiple serrated polyps.

## Conclusion

Despite a high serrated polyp count, only one-third of these CRCs demonstrated a *BRAF* V600E mutation, the molecular hallmark of the serrated neoplasia pathway, suggesting that though multiple serrated polyps act as a marker of an abnormal mucosa, the majority of CRC in these patients may arise through other mechanisms.
